# Addition of Resolvins D1 or E1 to Collagen Membranes Mitigates Their Resorption in Diabetic Rats

**DOI:** 10.3390/jfb14050283

**Published:** 2023-05-19

**Authors:** Michal Almogy, Ofer Moses, Nathan Schiffmann, Evgeny Weinberg, Carlos E. Nemcovsky, Miron Weinreb

**Affiliations:** 1 Department of Oral Biology, The Maurice and Gabriela Goldschleger School of Dental Medicine, Tel-Aviv University, Tel Aviv-Yafo 6997801, Israel; michal.almogy@gmail.com (M.A.); nathan@mail.tau.ac.il (N.S.); evgenywein@gmail.com (E.W.); 2 Department of Periodontology and Implant Dentistry, The Maurice and Gabriela Goldschleger School of Dental Medicine, Tel-Aviv University, Tel Aviv-Yafo 6997801, Israel; mosesofer@gmail.com (O.M.); carlos@tauex.tau.ac.il (C.E.N.)

**Keywords:** type 1 diabetes, collagen membrane, resolvins, RvD1, RvE1, medical device

## Abstract

Uncontrolled diabetes is characterized by aberrant inflammatory reactions and increased collagenolysis. We have reported that it accelerates the degradation of implanted collagen membranes (CM), thus compromising their function in regenerative procedures. In recent years, a group of physiological anti-inflammatory agents called specialized pro-resolving lipid mediators (SPMs) have been tested as a treatment for various inflammatory conditions, either systemically or locally, via medical devices. Yet, no study has tested their effect on the fate of the biodegradable material itself. Here, we measured the in vitro release over time of 100 or 800 ng resolvin D1 (RvD1) incorporated into CM discs. In vivo, diabetes was induced in rats with streptozotocin, while buffer-injected (normoglycemic) rats served as controls. Resolvins (100 or 800 ng of RvD1 or RvE1) were added to biotin-labeled CM discs, which were implanted sub-periosteally over the calvaria of rats. Membrane thickness, density, and uniformity were determined by quantitative histology after 3 weeks. In vitro, significant amounts of RvD1 were released over 1–8 days, depending on the amount loaded. In vivo, CMs from diabetic animals were thinner, more porous, and more variable in thickness and density. The addition of RvD1 or RvE1 improved their regularity, increased their density, and reduced their invasion by the host tissue significantly. We conclude that addition of resolvins to biodegradable medical devices can protect them from excessive degradation in systemic conditions characterized by high degree of collagenolysis.

## 1. Introduction

Diabetes mellitus is a metabolic disorder caused by a defect in insulin secretion, insulin action, or both, resulting in chronic hyperglycemia and interference in the metabolism of carbohydrates, fats, and proteins [1]. Chronic hyperglycemia is associated with many complications, such as neuropathy, nephropathy, retinopathy, cardiovascular disease, poor healing, and periodontal disease [2,3,4,5,6,7]. 

Most complications result from a global state of chronic, uncontrolled inflammation, associated with higher levels of inflammatory mediators, such as TNF-α, IL-1β, and IL-6, and greater numbers of immune cells, such as macrophages and neutrophils [3,5,8]. An increased presence of inflammation, as evidenced either by its cellular make-up or molecular repertoire, has been described in skin [9], gingiva and periodontal tissue [10], testes [11], retinae [6], blood vessels [7,12], kidneys [7], and bones [13] of diabetic humans or animals. As expected from an exaggerated, dysregulated inflammatory state, many of the diabetic complications are associated with the increased activity of collagenolytic enzymes, including MMPs (matrix metalloproteinases) and cathepsins [14], as documented in diabetic retinae [15], kidneys [16], tendons and intervertebral discs [17], blood vessels [18], periodontal ligament and gingiva [19], skin [20], etc. Hyperglycemia also induces oxidative stress and the production of reactive oxygen species (ROS) and advanced glycation end-products (AGEs). These, in turn, result in cellular damage and death (e.g., [21]) and impaired proliferation [22] and add greatly to the pathophysiology of diabetic complications [3,8,23].

Collagen, the major component of connective tissues, tendons, and ligaments, is a resorbable, naturally occurring protein, and has been incorporated into a variety of medical devices for many purposes. It is readily available from several animal sources, including bovine or porcine skin, tendons, and intestines, and can be easily combined with other biomaterials such as synthetic polymers (PLA and PLGA), hydroxyapatite, alginate, etc. Collagen-based biomaterials have been used in almost all possible physical states for many medical/dental applications. These include disc/meniscus/tendon repair [24], soft tissue augmentation in periodontal and dental implant treatments [25], bone augmentation around teeth and dental implants [26,27], wound healing improvements [28], delivery of biologics such as BMPs [29,30,31] or antibiotic/anti-inflammatory compounds [32], and delivery of stem/progenitor cells to diseased sites [33].

Due to the increased inflammation, ECM (extracellular matrix)-based devices face a more hostile environment after their implantation in diabetic animals or humans. A handful of studies have shown increased signs of inflammation (lymphocytes, macrophages, TNFα, and MCP-1) and increased activity of degrading enzymes around such devices implanted in diabetic animals [34,35,36].

Barrier membranes made of various biomaterials are a sub-type of medical devices and are applied regularly in regenerative procedures in periodontics, orthopedics, and maxillofacial surgery [37,38,39,40]. They provide structural support and tissue separation. The most commonly used membranes are resorbable, made of collagen (type 1 or 3), and their resorption is mediated by the collagenolytic activity of host cells [41,42]. Our previous studies have found that collagen membranes undergo an accelerated degradation in diabetic animals, accompanied by an inflammatory reaction [43,44,45,46]. 

In recent years, a group of physiological anti-inflammatory agents, called specialized pro-resolving lipid mediators (SPMs), such as lipoxins, resolvins, maresins, and protectins, have been identified and tested for their ability to combat local and systemic inflammatory conditions. SPMs are autacoids, synthesized from omega-3 polyunsaturated fatty acids derivatives. Resolvins include E series resolvins (RvE1 and RvE2), derived from eicosapentaenoic acid (EPA), and D series resolvins (RvD1-4), derived from docosahexaenoic acid (DHA). Protectins and maresins are also derivatives of DHA [47,48]. SPMs are produced locally at the site of inflammation and reduce or prevent polymorphonuclear cell infiltration, vascular permeability, and the release of pro-inflammatory molecules. As a result, there is a decrease in the release of MMPs from fibroblasts and macrophages. These combined effects result in inflammation resolution [49,50,51]. In chronic inflammation, such as that present in diabetes, resolvin production is low, even in acute inflammatory circumstances [52]. 

Systemic and local administration of SPMs to treat various inflammatory conditions has been extensively studied. For instance, RvE1 and RvD1 prevented experimental colitis in mice [53]. Systemic administration of resolvins also decreased inflammatory reactions in different diabetic animal models [54,55], such as periodontitis and colitis, by limiting the activity of neutrophils and macrophages and thus inhibiting collagenolysis and promoting bone and tissue repair [4,54].

Local delivery of SPMs has been investigated in vivo via different medical devices. Positive effects of resolvins delivered via various dressings on wound healing were found in mice. Other studies have shown beneficial effects of local resolvin delivery in surgical models of vascular injury in rats and rabbits [56,57,58].

In dental tissues, the application of RvE1 directly to the injured pulp of rat molars improved and accelerated the organization of the dentin bridge and decreased the likelihood of pulp necrosis by attenuating secretion of TNF-α and IL-1β [59]. In addition, the local application of RvE1 to sites of periodontitis, induced in rabbits, inhibited bone loss and tissue damage [60]. 

There is currently no study, which has investigated the effects of incorporation of resolvins on the fate of the medical devices that carry them. In this study, we measured the in vitro release of RvD1 and assessed the impact of the release of RvD1 or RvE1 in vivo from a medical device (collagen membrane) on its resorption in diabetic rats.

## 2. Materials and Methods

### 2.1. Collagen Membrane Preparation and Labeling

Bio-Gide^®^ collagen membranes (Geistlich Pharma, Wolhusen, Switzerland) were cut into 6 mm diameter discs using a disposable biopsy punch. All discs were labeled with 3 mg/mL biotinamidohexanoic acid N-hydroxysuccinimide ester (MERCK, Darmstadt, Germany) for 1 h at room temperature, washed three times with Dulbecco’s phosphate-buffered saline (DPBS, calcium/magnesium- free, pH 7.4, Biological Industries, Beit-Haemek, Israel) for 12 h and dried overnight.

### 2.2. Resolvin Incorporation

Resolvin D1 or resolvin E1 (Cayman chemical, Ann Arbor, MI, USA) (100 or 800 ng per disc in 20 µL of an ethanol solution) was added dropwise to the discs using a micropipette. Discs were then lyophilized at −100 °C and 1.1 bar for 24 h and kept at −80 °C.

### 2.3. Kinetic Release Study of RvD1

The release kinetics of RvD1 were studied in collagen membrane (CM) discs of 6 mm diameter divided into two equal groups, biotinylated and non-biotinylated, using a resolvin D1 ELISA kit (Cayman Chemical). Within each group, either ethanol, 100 ng RvD1, or 800 ng RvD1 were added to the disks as described above. After lyophilization, discs were immersed in 3 mL of DPBS at 37 °C. Half of the buffer was collected at the following time points: 2, 5, 10, 24, and 192 h. In the 800 ng group, we added two more time points: 72 and 96 h. Every time a sample was collected, the buffer was replenished with an equal amount of fresh DPBS at 37 °C. The concentration, and hence the content, of RvD1 in each sample was calculated and a cumulative release curve was constructed for each group using the mean and se of four replicates.

### 2.4. Animals

The in vivo study included 54 three-month-old male Wistar rats weighing 250–300 g (Envigo, Jerusalem, Israel). The animals were divided into six equal groups of nine rats each:

1. Normoglycemic (control) + disc/ethanol;
2. Diabetic + disc/ethanol;
3. Diabetic + disc/100 ng RvD1;
4. Diabetic + disc/800 ng RvD1;
5. Diabetic + disc/100 ng RvE1;
6. Diabetic + disc/800 ng RvE1.

This study was approved by the institutional animal care and use committee of the Faculty of Medicine at Tel-Aviv University, number 01-21-029.

### 2.5. Induction of Diabetes

In the diabetic groups, type-1-like diabetes was induced through a single intraperitoneal injection of streptozotocin (STZ, 65 mg/kg of body weight, Sigma-Aldridge, St. Louis, MO, USA) dissolved in a citrate buffer (0.01 M, pH 4.5). Blood glucose levels were measured twice a week via a drop of blood from the tail vein using a glucometer (Accu-Check, Roche Diagnostics, Basel, Switzerland). Rats with a glucose level higher than 250 mg/dL were considered diabetic. In the control group, a single intraperitoneal injection of citrate buffer (0.01 M, pH 4.5) was administered.

### 2.6. Surgery and Tissue Handling

Surgery was performed 7 days after diabetes induction. Animals were anesthetized using ketamine chlorhydrate 2% (90 mg/kg) and xylazine (10 mg/kg) administered by an i.p. injection. All animal surgeries were performed by the same surgeon. The dorsal part of the scalp was shaved and aseptically prepared. A sub-periosteal pouch over the skull was generated surgically and a single collagen membrane disc was placed in each pouch. Soft tissues were repositioned and sutured with resorbable sutures. Wound healing was monitored twice a week. Three weeks after surgery, animals were sacrificed by a ketamine/xylazine overdose and carbon dioxide (CO_2_) and the membrane with its surrounding soft and hard tissues was collected, fixed in buffered paraformaldehyde (4%), decalcified for 7 weeks with EDTA (10%), and embedded in paraffin for histological evaluation.

### 2.7. Histology

Sagittal 5 µm sections were stained with horseradish peroxidase-conjugated streptavidin (Sigma-Aldridge) to detect the remaining biotinylated collagen from the original membrane. Images of stained sections were obtained using an Aperio Versa 200 system (Leica Biosystems Imaging, Wetzlar, Germany). The collagen membrane thickness and density were calculated using ImageJ software (NIH, Bethesda, MD, USA). 

The membrane thickness was measured at 10–15 sites along its entire length and a mean thickness was calculated for each section (animal). 

In addition, we introduced three thickness-related parameters for membrane regularity:

1. Coefficient of variation (CV) of the absolute membrane thickness;
2. Mean thickness (as percentage of the maximal membrane thickness within each membrane);
3. Standard deviation (sd) of mean percentage of maximal membrane thickness.

For density measurements, a square region of interest (ROI) with a side equal to the membrane thickness was constructed over the thickest portion of the membrane and the percent area occupied by original collagen was measured. This ROI was moved along the membrane without overlap and measurements were obtained repeatedly.

Original collagen identification and validation were performed using image-processing methods via pseudo-colored overlays on top of the biotin-avidin-HRP image.

A mean residual membrane area (as a % of the ROI area) was calculated for each section (animal). In addition, we introduced three parameters for membrane density regularity that are independent of the absolute density of each membrane:

1. Coefficient of variation (CV) of membrane density along the entire membrane;
2. Mean density (as a percentage of the maximal membrane density within each membrane);
3. Standard deviation (sd) of mean density (as a percentage of maximal membrane density).

Adjacent sections were stained with hematoxylin and eosin for general inspection, assessment of inflammation, and penetration of host tissue into the membrane. A few additional representative sections were stained for elastin fibers using the Verhoeff procedure.

### 2.8. Statistical Analysis

The results are stated as mean ± se A comparison between group means was performed using an analysis of variance and, wherever it was significant (*p* < 0.05), followed by LSD post hoc tests between specific groups.

## 3. Results

### 3.1. RvD1 Kinetic Release Study

**Figure 1:**
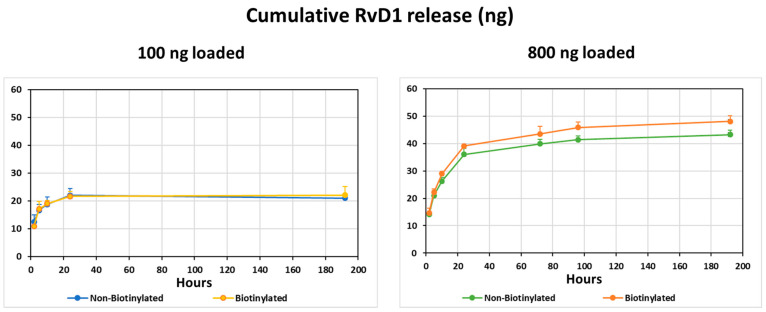
RvD1 release from biotinylated or non-biotinylated collagen membrane discs in two sub-groups: 100 ng and 800 ng of RvD1. For each time point, the mean and se of four replicates is shown.

RvD1 was added to collagen membrane discs (with or without pre-labeling with biotin) and its release curves in the different groups are presented in Figure 1. 

Collagen discs, to which 100 ng RvD1 were incorporated, released a total of 20.9 ± 2.2 ng (mean ± sd) of RvD1 from non-biotinylated discs and 22.0 ± 2.3 ng from biotinylated ones at 192 h. Both groups reached a plateau after around 24 h. Collagen discs containing 800 ng RvD1 released a total of 43.3 ± 1.4 ng from non-biotinylated discs and 48.1 ± 3.9 ng from biotinylated discs at 192 h, without a visible plateau. The results show that, firstly, biotin-labeling of the membranes had a limited effect on RvD1 release, and in the 800-ng group, RvD1 release was slightly increased after biotinylation. Secondly, the discs in the 800 ng group released more RvD1 than those in the 100 ng group, with a plateau after more than 192 h. These data show that collagen membranes can absorb RvD1 and release it over time with different kinetics. 

### 3.2. Animals, Surgery, and Autopsy

Out of forty-five STZ-injected rats, only forty presented glucose levels higher than 250 mg/dL after three days and remained hyperglycemic for the entire experiment. One rat died during surgery and in another, the membrane could not be found at autopsy. One additional rat developed an infection at the site of implantation and three membranes in the control group were located outside the periosteal pouch, and all of these were excluded from the analysis.

### 3.3. Qualitative Evaluation

**Figure 2:**
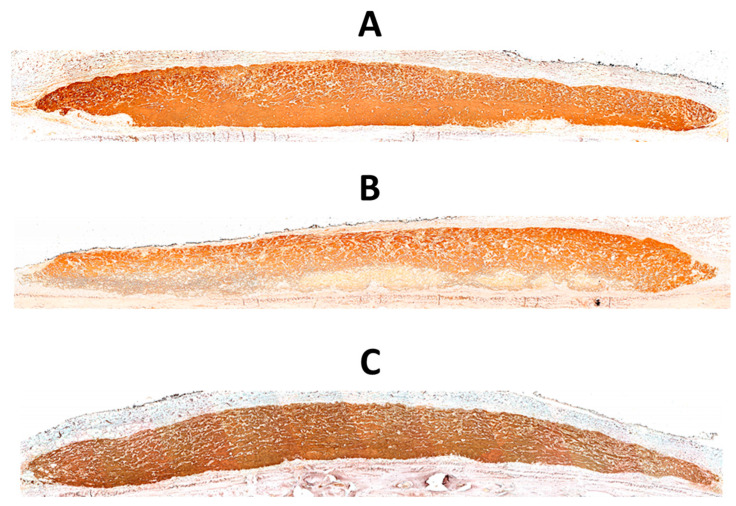
Histologic view (×50) of biotin-avidin-HRP-stained collagen membrane (brown-red) in a rat from the control group (**A**), the diabetic group (**B**), and the 800 RvD1 group (**C**).

Figure 2 shows histologic views of biotin-avidin-HRP-stained collagen membranes. The ethanol-treated membrane from the control (normoglycemic) group exhibits a higher biotin-avidin-stained area, i.e., a denser core (Figure 2A). In contrast, the ethanol-treated membrane from the diabetic group is thinner and exhibits larger gaps, a lower density of biotin-avidin staining, and a greater variability in thickness (Figure 2B). However, the membrane from the diabetic group, to which 800 ng of RvD1 were added (Figure 2C), has fewer and smaller voids than the diabetic membrane and a greater uniformity in thickness, thus resembling the control membrane.

### 3.4. Quantitative Measurements of Membrane Thickness

**Figure 3:**
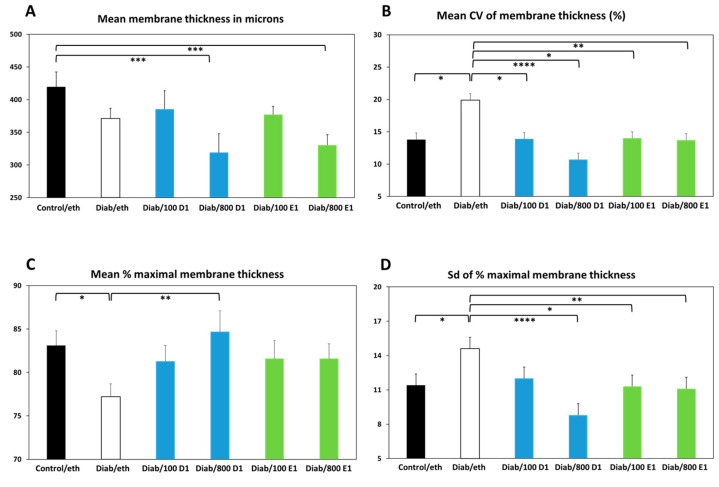
(**A**) Mean membrane thickness in microns; (**B**) mean CV of membrane thickness in %; (**C**) mean % of maximal membrane thickness; (**D**) sd of % maximal membrane thickness. Control = normoglycemic; eth = ethanol; Diab = diabetic; 100 D1 = 100 ng RvD1; 800 D1 = 800 ng RvD1; 100 E1 = 100 ng RvE1; 800 E1 = 800 ng RvE1. * *p* < 0.05; ** *p* < 0.01; *** *p* < 0.005; **** *p* < 0.001.

The mean thickness of CMs in the control group was 419.5 ± 23.1 microns, whereas in the diabetic group it was 371.1 ± 15.6 microns, without statistical significance (Figure 3A). CMs in the 800 ng groups (both RvD1 and RvE1) were significantly (*p* < 0.005) thinner than those in the control group, with mean membrane thicknesses of 319.2 ± 28.7 and 330.3 ± 16.1 microns, respectively. The reason for this observation is not yet clear.

In addition to membrane thickness, we assessed three parameters indicating membrane regularity, the first being the variability (coefficient of variation) in the membrane thickness (shown in Figure 3B). The results show greater variability in thickness in CMs of the diabetic group compared with controls. Membrane thickness regularity was restored to control values in membranes treated with either RvD1 or RvE1 (both 100 ng and 800 ng). 

The second parameter of membrane regularity is the mean percentage of maximal membrane thickness throughout its length (Figure 3C). The collagen membranes in the control group had a mean thickness that was 83.1 ± 1.7% of the maximal thickness, compared with 77.2 ± 1.5% in the diabetic group (*p* < 0.05), pointing to the existence of more thinner zones in the latter. All treatments of the membrane increased the membrane thickness uniformity, with a significant effect in the group treated with 800 ng of RvD1 (84.7 ± 2.4%, *p* < 0.01).

The last parameter applied to measure the membrane uniformity was the mean standard deviation of the thickness as a percentage of the maximal thickness (Figure 3D). Membranes from the diabetic group showed a significantly larger variability (higher sd) in thickness compared with membranes from control animals (*p* < 0.05). All treatments reduced the membrane thickness irregularity, with statistically significant effects in membranes treated with 800 ng of RvD1, 100 ng of RvE1, and 800 ng of RvE1.

The data of the last three parameters clearly indicate that, regardless of the absolute thickness of the membranes, those from the diabetic group were significantly more irregular in width, with many thinner areas, and that addition of RvD1 or RvE1 largely reduced all the irregularities in thickness to the control values.

### 3.5. Quantitative Measurements of Membrane Density

**Figure 4:**
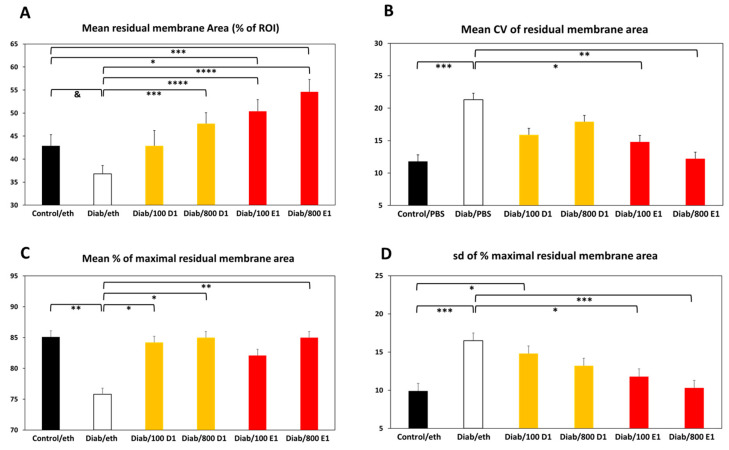
(**A**) Mean residual membrane area (% of ROI); (**B**) mean CV of residual membrane area (in %); (**C**) mean % of maximal residual membrane area; (**D**) sd of % maximal residual membrane area. Control = normoglycemic; eth = ethanol; Diab = diabetic; 100 D1 = 100 ng RvD1; 800 D1 = 800 ng RvD1; 100 E1 = 100 ng RvE1; 800 E1 = 800 ng RvE1. & *p* = 0.06; * *p* < 0.05; ** *p* < 0.01; *** *p* < 0.005; **** *p* < 0.001.

Firstly, the area occupied by the residual membrane as a percentage of the ROI was calculated. As shown in Figure 4A, the residual area of the membranes in the diabetic group was significantly smaller than that in the control group (*p* = 0.06). Addition of 100 ng RvD1 to the membranes increased their residual area to the control levels and all the other treatments significantly increased the residual area (vs. the diabetic group), even beyond the control levels.

Next, we used three parameters to assess the regularity of membrane density. Figure 4B shows that the variability (as a coefficient of variation) in the membrane density was significantly greater in the diabetic group (*p* < 0.005). All treatments reduced the density variability, with significant effects in the groups treated with 100 and 800 ng RvE1.

Within the same membrane, the mean density as a percentage of its maximal density represents another uniformity parameter. Figure 4C shows that membranes from the diabetic group contained many areas where the collagen density was lower than the maximal density in the same membrane. The mean relative density in the control group was 85.1 ± 2.8% and it was reduced to 75.8 ± 2.8% in the diabetic group (*p* < 0.01). All treatments reduced the variability in the relative membrane density to control levels, with significant effects in the groups treated with 100 ng of RvD1, 800 ng of RvD1, and 800 ng of RvE1.

The last parameter of membrane uniformity/regularity is the sd of the percentage of maximal density in the different parts of the membrane. Figure 4D shows a greater variability (higher sd) in the membrane density in the diabetic group compared to the control group (*p* < 0.005). All treatments reduced the variability in the density within the membranes, with significant effects in the groups treated with 100 and 800 ng of RvE1.

### 3.6. H&E Staining Findings

**Figure 5:**
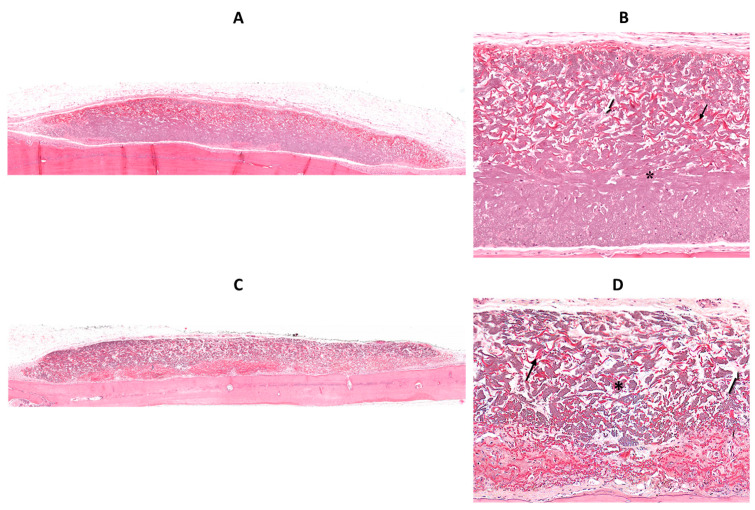
(**A**,**C**): Histologic view (×40) of an H&E-stained collagen membrane in a rat from the control group and diabetic group, respectively. (**B**,**D**) show a higher magnification (×125) of these membranes. * old (original) collagen fibers (purple), black arrows—new elastic fibers, white arrows—new collagen fibers.

**Figure 6:**
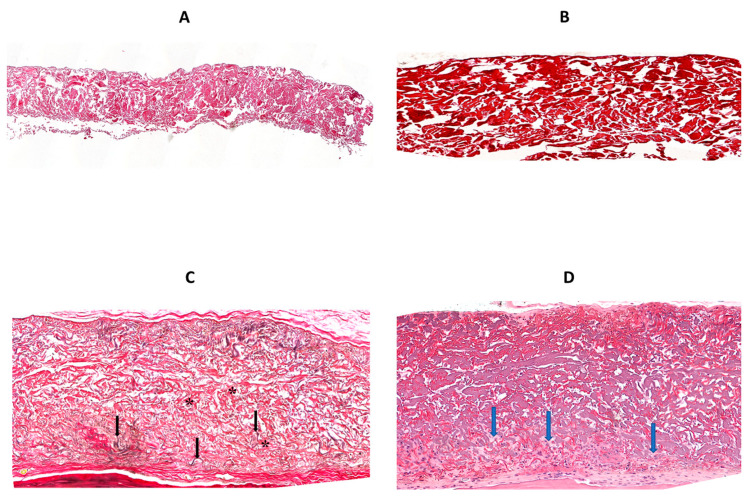
(**A**,**B**): Histologic view of a non-implanted membrane stained with H&E (×40) and elastin (×125), respectively. (**C**) is a section of an implanted membrane stained for elastin (×125) showing positive, purple, undulating fibers (arrows) among old (original) collagen fibers (*). (**D**) Section of an implanted membrane stained for H&E showing new collagen fibers (blue arrows) (×125).

Membranes from the diabetic group (Figure 5C) were thinner, irregular in thickness, and contained greater gaps, compared with those from the control group (Figure 5A). It is noteworthy that the control membranes contained less than 45% of the original collagen by area at sacrifice (Figure 4A). Most, but not all, of the area not occupied by original collagen stems from voids, which are present upon manufacture (see Figure 6A of a non-implanted membrane stained with H&E).

Implanted membranes contained three types of fibers; the majority of the membrane volume contained thick, purple collagen fibers (* in Figure 5B,D) that were part of the original membrane. In addition, numerous red, thin, undulating fibers were found within the gaps between the original collagen fibers (black arrows in Figure 5B,D, H&E stained). These could theoretically be either elastic fibers from the original membrane, new elastic fibers that were deposited within the gaps post-implantation, or new collagen fibers. These undulating fibers are not present in the original membrane (Figure 6A (H&E) or Figure 6B (elastin stain)) and are positive for elastin staining in the implanted membrane (Figure 6C) and therefore are newly formed elastic fibers located among the original collagen fibers.

The third type of fibers present in the transplanted membranes is new collagen fibers that had been deposited within the gaps post-implantation (blue arrows in Figure 6D).

**Figure 7:**
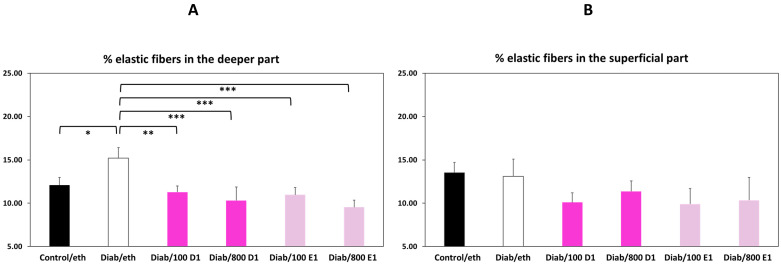
Percentage area occupied by new elastic fibers in the superficial (**A**) and deeper (**B**) part of the membrane. Control = normoglycemic; eth = ethanol; Diab = diabetic; 100 D1 = 100 ng RvD1; 800 D1 = 800 ng RvD1; 100 E1 = 100 ng RvE1; 800 E1 = 800 ng RvE1. * *p* < 0.05; ** *p* < 0.01; *** *p* < 0.005.

Finally, we quantified the area occupied by the new elastic fibers within the implanted membranes as a measure of host cell penetration. The membrane was divided into two equal parts: a deeper one (closer to the calvaria) and a superficial one (under the periosteum, away from the underlying bone). There were no significant differences between the groups in the percentage area occupied by elastic fibers in the superficial part (Figure 7A). In contrast, there were significantly more new elastic fibers in the deeper part of the membranes from the diabetic group, compared with the control group (Figure 7B). All treatments of the membranes significantly reduced the presence of new elastic fibers within the gaps of the deeper part to the value of the control group.

## 4. Discussion

The aim of this study was to evaluate the effect of incorporating RvD1 and RvE1 on collagen membrane (CM) resorption in diabetic rats, in which there is a significant background of chronic inflammation.

The first question was whether our medical device (CM) can absorb and release resolvins over a meaningful period. The in vitro results showed that the answer is positive, since RvD1 (the only resolvin that can be measured at present) was released over 1–8 days (at least) depending on the amount loaded. The CM incorporated with 100 ng of RvD1 reached a plateau at around twenty-four hours and those with 800 ng of RvD1 continued to release and did not reach a plateau until after eight days. These results are similar to other studies that tested the release kinetics of RvD1 over time from other biodegradable devices [61,62].

The second issue was whether immersion of the membrane in biotin (needed to identify the original collagen after sacrifice) interferes with resolvin adsorption and release. We found this not to be the case, since RvD1 release from biotin-labeled CMs was the same in the 100 ng group and even greater in the 800 ng group, compared with non-biotinylated CMs. These results may be interpreted to mean that our collagen membranes serve as a slow-release device and deliver significant amounts of resolvin to the implantation site.

Our previous studies [43,44,45,46] clearly indicate that CMs undergo a more rapid resorption when implanted under the scalp of hyperglycemic rats. This is associated with an increased presence of macrophages and other immune cells, and an increased production of pro-inflammatory cytokines such as IL-6 and TNFα. Immersion of the membranes in tetracycline or hyaluronic acid significantly reduced their excessive degradation associated with hyperglycemia. Thus, we hypothesized that the delivery of resolvins (anti-inflammatory molecules) will curb the inflammatory response and protect the collagen devices from excessive degradation. 

The first main finding of the histologic analysis in this study is that CMs implanted in diabetic rats were thinner, more irregular in thickness and density, and more porous, compared with those from normoglycemic animals. In addition, invasion of the CMs by the host tissues was more prominent in the diabetic group, as evidenced by a greater presence of new connective tissue fibers. Since host collagenolytic enzymes degrade collagen within the implanted CM, these data are in full agreement with our previous reports, which indicate increased collagen membrane degradation in diabetic animals. From a clinical point of view, these changes (i.e., membranes that are thinner, irregular, and porous) imply a deterioration in their tissue barrier function.

The second main finding of the results in our study indicates that addition of RvD1 or RvE1 to the CMs protected them from excessive resorption and improved their regularity and density. Except for absolute thickness, addition of RvD1 or RvE1 reversed all diabetes-induced changes in membrane thickness and density regularity, often beyond the control values. Furthermore, RvD1 and RvE1 mitigated the invasion of host tissues into the membrane, as evidenced by a lower number of elastic fibers that had been deposited within the CMs (Figure 7B). Interestingly, there was a quasi-dose response in the effects of RvD1 on membrane thickness (Figure 3B–D) and in the effects of RvE1 on membrane density (Figure 4A–D). These data indicate that although CMs from the resolvin groups were somewhat thinner, they were much denser and significantly more regular in thickness and density, even beyond the control CMs. These changes significantly improve collagen device functionality both in dentistry and in medicine by prolonging their survival in host tissues. As such, addition of RvD1 or RvE1 to CMs is a counter-measure against excessive resorption of medical devices in a high-risk situation such as hyperglycemia.

Although inflammation parameters were not measured in the current study, we speculate that the release of resolvins from implanted CMs inhibited local inflammation and consequent collagenolysis, thereby protecting the CMs from degradation by the host. This hypothesis is based on two lines of evidence in existing data. Firstly, under diabetic conditions, there are increased levels of inflammation and thus secretion of pro-inflammatory mediators such as IL-1β, IL-6, and TNFα [3,5,8]. This is true for many organs, including the periodontium, for CMs implanted in diabetic animals (our studies [43,44,45,46]) and for other degradable devices implanted in diabetic animals [34,35,36]. Increased lysis of various tissues in diabetics accompanies the exaggerated inflammation [14,15,16,17,18,19,20,63,64,65]. By inference, this is corroborated by our findings that addition of tetracycline (anti-collagenolytic) and hyaluronic acid (anti-inflammatory) to the membranes mitigated their excessive resorption in diabetic rats (our studies [43,44,45,46]). 

Secondly, resolvins are natural molecules that can dampen or “resolve” inflammatory responses [47]. In vitro, Resolvins can reduce the pro-inflammatory activity of neutrophils and macrophages [56,66] and decrease IL-6 and TNFα production in macrophages, due to a switch in polarization from a pro-inflammatory phenotype (M1) to an anti-inflammatory phenotype (M2) [67]. In addition, resolvins can reduce NF-κB activation in rat arterial smooth muscle cells, which, in turn, reduces the inflammatory response to arterial injuries [68].

In vivo studies have shown that systemic administration of SPMs could reduce the inflammatory response in various animal models [47,48,53,54,69], including inflammation caused by diabetes [55,70]. Local injuries (such as skin wounds, vascular injuries, dental injuries, etc.) that result in inflammation can also be treated by delivering SPMs locally to the injured area or via a variety of implantable medical devices [56,57,58,59,60,61,66,68,71,72,73]. 

This combined published evidence can support our hypothesis and suggests a mechanism of action in our experimental model, namely that resolvins released from the implanted medical device dampen inflammation and collagenolysis within and around it.

Our data indicate that SPMs are able not only to dampen inflammation in tissues located adjacent to the point of delivery [56,57,58,61,66,68,71,72,73], but also to protect medical devices that carry them from being resorbed prematurely in circumstances characterized by a high degree of collagenolysis. Preventing the resorption of bioresorbable medical devices prolongs their survival and improves their functionality in terms of both separating and supporting tissues and their ability to deliver biologics.

Further research is needed to determine how the local delivery of resolvins affects the cellular and molecular components of the local inflammatory response and collagenolytic activity around and within collagen devices implanted in diabetic rats.

## 5. Conclusions

Collagen membranes are able to adsorb and release resolvins (RvD1 and RvE1) in vitro over at least a few days. In vivo, addition of these anti-inflammatory molecules protects collagen membranes from exaggerated resorption in diabetic rats, most likely by attenuating the inflammation and collagenolytic activity around them. Mitigating the resorption of ECM medical devices prolongs their survival in hostile in vivo circumstances and improves their functionality.

## Data Availability

Not applicable.
